# Rethinking the syndemic of tuberculosis and dysglycaemia: a Kenyan perspective on dysglycaemia as a neglected risk factor for tuberculosis

**DOI:** 10.1186/s42269-023-01029-6

**Published:** 2023-04-13

**Authors:** Cheryl Kerama, David Horne, Jane Ong’ang’o, Omu Anzala

**Affiliations:** 1 Centre for Respiratory Diseases Research, Kenya Medical Research Institute (CRDR-KEMRI), Past Government Chemist, Opposite Diabetes Clinic, Nairobi, Kenya; 2 Division of Medical Microbiology and Immunology, Kenya AIDS Vaccine Initiative-Institute for Clinical Research (KAVI-ICR), Nairobi, Kenya; 3 Division of Pulmonary and Critical Care and Sleep Medicine, University of Washington, Seattle, WA USA; 4 School of Medicine, Faculty of Health Sciences, University of Nairobi, Nairobi, Kenya

**Keywords:** Tuberculosis, Dysglycaemia, Diabetes, Prediabetes, HIV, END TB 2035, NCDs

## Abstract

**Background:**

The END TB 2035 goal has a long way to go in low-income and low/middle-income countries (LICs and LMICs) from the perspective of a non-communicable disease (NCD) control interaction with tuberculosis (TB). The World Health Organization has identified diabetes as a determinant for, and an important yet neglected risk factor for tuberculosis. National guidelines have dictated testing time points, but these tend to be at an isolated time point rather than over a period of time. This article aims to give perspective on the syndemic interaction of tuberculosis and dysglycaemia and how the gaps in addressing the two may hamper progress towards END TB 2035.

**Main text:**

Glycated haemoglobin (HbA1C) has a strong predictive association with the progression to subsequent diabetes. Therefore, screening using this measure could be a good way to screen at TB initiation therapy, in lieu of using the random blood sugar or fasting plasma glucose only. HbA1C has an observed gradient with mortality risk making it an informative predictor of outcomes. Determining the progression of dysglycaemia from diagnosis to end of treatment and shortly after may offer information on the best time point to screen and follow-up. Despite TB and Human Immunodeficiency Virus (HIV) disease care being free, hidden costs remain. These costs are additive if there is accompanying dysglycaemia. Regardless of receiving TB treatment, it is estimated that almost half of persons affected by pulmonary TB develop post-TB lung disease (PTLD) as an outcome and the contribution of dysglycaemia is not well described.

**Conclusions:**

Establishing costs of treating TB with diabetes/prediabetes alone and in the additional context of HIV co-infection will inform policy makers on what it takes, financially, to treat these patients and subsidize dysglycaemia care. In Kenya, cardiovascular disease is only rivalled by infectious disease as a cause of mortality, and diabetes is a well-described risk factor for cardiac disease. In poor countries, communicable diseases are responsible for majority of the mortality burden, but societal shifts and rural–urban migration may have contributed to the observed increase of NCDs.

## Key points

- The END TB 2035 goal has a long way to go in LICs and LMICs from a non-communicable disease (NCD) control perspective.
- The WHO has established diabetes as an important and neglected risk factor for TB.
- Poverty is a barrier to healthcare.
- Globally, in 2019, almost half of all people with TB and their households faced catastrophic costs (defined as total costs equivalent to > 20% of annual household income) and rising up to 80% for those households with a member having drug-resistant TB.
- Though TB and HIV healthcare is ‘free’, hidden costs remain which have far-reaching socio-economic effects on households.
- Even with curative treatment, nearly half of persons affected by pulmonary TB develop post-TB lung disease (PTLD) as an outcome and the contribution of dysglycaemia is not well described.
- While the relationship between diabetes and TB is well described, the work on the dysglycaemia spectrum in between is not as well described.
- Funding for TB research has fallen below less than half of target despite most low to low/middle income (LMCs and LMICs) being heavily reliant on international donor funding.

## Background

The End TB Strategy set an ambitious goal of a 90% reduction in the number of persons affected by TB and 95% reduction in the number of deaths due to TB by 2035. As this milestone draws near, these TB targets are unlikely to be met. Major reductions in TB morbidity and mortality can only be achieved if TB diagnosis, treatment and prevention services are provided within the context of progress towards universal health coverage (UHC), and if there is multisectoral action and accountability to address the broader determinants that influence the TB epidemic and their socio-economic impact (WHO 2020).This statement means that without multi-stakeholder cooperation to address non-communicable diseases among the determinants affecting TB, this milestone may be out of reach. One such determinant is diabetes. The WHO has established diabetes as an important and neglected risk factor for TB (WHO 2020).

**Fig. 1:**
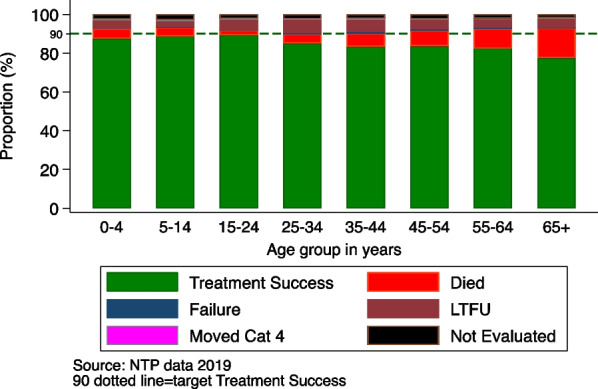
Treatment outcome by age groups for TB patients notified in 2019 in Kenya (Program-NTLLD-P NTLaLD 2021)

Ischaemic heart disease (IHD), stroke, TB and diabetes mellitus are among the top 10 in the list of disability life-adjusted years (DALYs) according to the estimates from 2019 (The Global Health Observatory 2021). Given the disability and mortality burden associated with tuberculosis and diabetes, it may be time to begin zooming in a closer lens on more effective strategies to manage these two epidemics. Based on the 2021 epidemiological report by the Kenya National TB Program (NTP), the treatment success across the age groups of TB patients declines from 34–44 years to the 65+ years of age. It could be hypothesized that given the risk of diabetes increases from age 35, there could be a possibility that the deaths in this age group are related to having dysglycaemia as a comorbidity. The oldest (65+ years) proportional wise comparison with other age groups, have a high proportion of deaths (see Fig. 1) (Program-NTLLD-P NTLaLD 2021). There is need to address TB in a holistic fashion incorporating screening for non-communicable disease in the elderly who often times, have other comorbidities like diabetes and hypertension which increase the risk of dying during TB treatment (Program-NTLLD-P NTLaLD 2021).

Individuals living with diabetes mellitus (DM) have three times the risk of developing TB and there are now more individuals with TB-DM comorbidities than those with TB-HIV co-infection (Jeon and Murray 2008; Ronacher et al. 2015).The effects of DM on TB have been well described including atypical presentation both clinically and on chest radiograph (CXR) and higher likelihood of poor treatment outcomes like relapse, treatment failure and even death (Dooley and Chaisson 2009). Much less is known about the impact of non-DM dysglycaemia (such as prediabetes and transient hyperglycaemia) on TB risk (Liu et al. 2021). Other dysglycaemia subtypes have been described in patients with active TB and have a TB risk profile similar to diabetes, but may not be recognized in the clinic setting due to the way DM screening is performed (Liu et al. 2021; Alkabab et al. 2021). It is clear that more robust interventions, than merely describing diabetes in relation to TB and TB/HIV co-infection, are needed.

This topic is important because TB, prior to the COVID-19 pandemic, was the leading cause of death from a single infectious agent (MacNeil et al. 2020). Its interaction with diabetes is clearly described in terms of poor outcomes. More recent work has described non-DM entities (erratic glycaemic instability, transient hyperglycaemia) that are in the spectrum of dysglycaemia. They make up a component that is not yet fully diabetes but have similar risk profiles as frank diabetes itself. This means that lack of careful surveillance of the entire spectrum of dysglycaemia in relation to TB may compromise our efforts to curb the disease by 2035 as envisioned. Further to this, despite curative treatment, a chronic disease of the lungs, post-tuberculosis lung disease (PTLD) occurs in nearly half of persons treated for TB and the contribution of dysglycaemia to this phenomenon is not well described.

The time is nigh to write about this topic because there are currently more people living with TB and diabetes than there are living with TB and HIV, a more historically traditional risk factor which has received much attention. The END TB 2035 goal will require more than just diagnosis and treating TB, and more than giving TB preventive therapy (TPT). It needs more focus on multi-sectorial approaches that involve stakeholders from the non-communicable disease (NCD) sector as well as financial partners to reduce both the reciprocal effect of NCDs, more specifically dysglycaemia, and the socio-economic hardship occasioned by the catastrophic costs of TB on households.

Furthermore, from a viewpoint of TB control, contacts of persons with pulmonary TB and dysglycaemia (whether diabetes or prediabetes) have been found to be at increased risk of being QuantiFERON (QFT)-positive at baseline or at month six of treatment (Arriaga et al. 2021). Heightening of surveillance on this category of close contacts could improve gains towards control of TB, ultimately taking us closer to the END TB 2035 goal. However, how would we focus on such contacts if we do not aggressively tackle the issue of screening and follow-up of TB patients for dysglycaemia.

This scientific article will focus on the neglected risk factor and the spectrum of dysglycaemia, in terms of global, regional and local disease burden, the body of work that has been done so far in this area of work with a global snapshot and an emphasis on Kenya and Africa in terms of the reciprocal relationship of TB and, the socio-economic burden of the trio of TB,TB/HIV co-infection and dysglycaemia, the funding situation in the LICs and LMICs and the ultimate progress on the END TB 2035 goal as well as thoughts on how we should change strategy if we have any hope of achieving this goal.

## Main text

### Interplay of TB and diabetes: Global snapshot with emphasis on Africa and Kenya

Baker and his group in their systematic review of the impact of DM on anti-tuberculosis treatment outcomes found an increased risk of mortality of at least 89% during anti-tuberculosis treatment and a 3.9 times increased risk for TB relapse after successful completion of treatment (Baker et al. 2011). Bi-directional screening of TB and DM yields high TB prevalence among patients with DM, ranging from 1.7% to 36% which increased with rising TB prevalence in the population. The converse action; that is, the screening of patients with TB for DM has similarly yields a high DM prevalence, ranging between 1.9 and 35% (Jeon et al. 2010). Despite guidance from the World Health Organization (WHO), most facilities in sub-Saharan Africa are still not screening TB patients for DM, due to cost, perceived complexities and lack of a treatment infrastructure for those who screen positive (Adepoyibi et al. 2013; Harries et al. 2015; World Health O et al. 2011). Transient hyperglycaemia caused by TB disease or medication, and similarities in symptom presentation, such as weight loss, further complicate the process (Owiti et al. 2017). A very compelling body of work in terms of a pragmatic approach to this syndemic touches on the urgent need for clinical management strategies and more research evidence on the choice and dose of different anti-diabetes medication and effective medical therapies to reduce cardiovascular risks (statins, anti-hypertensives and aspirin) for primary care health workers. They further highlight that significant investment in health system strengthening and integration may be needed to prevent these at risk patients being lost to care at the end of TB treatment (Crevel and Critchley 2021). Most of the work done in TB and PWDM in Africa has revolved around prevalence with a predominantly cross-sectional study design. In Kenya, one study was done in a rural setting and another in an urban setting in relation to dysglycaemia and TB. The study in the rural setting found that there was 5% prevalence of DM and 37.5% of prediabetes, duration of follow-up was up to 4 months with a median of 8 weeks. Associations found that there was increased odds of DM with advancing age > 40 years and a family history of DM (Owiti et al. 2017). The urban setting study was done in Nairobi in two hospitals and found a 25.8% and 10.5% rate of impaired fasting glycaemia and diabetes, respectively. This study did not find an association between HIV and dysglycaemia (Amayo and Mutai 2018). Contrastingly, a recent hospital based study in Ethiopia found that HIV co-infection and family history of DM were independent risk factors for DM in TB patients (Tulu et al. 2021). More research is needed in Africa-based populations to determine the influence of HIV on dysglycaemia in the background of TB infection.

In Tanzania, Mabula et al. who conducted their work in Moshi, Kilimanjaro, confirmed the work of the group in Kenya that found odds of DM in TB increase with a family history of the same and advancing age > 40 years. This study did not evaluate for prediabetes but found that there was 9.2% rate of DM (Mabula et al. 2021).

In Mali, a study found that the rate of impaired fasting glycaemia was 8.5% and DM was 5.5% (Diarra et al. 2019).

### Socio-economic burden of the TB and dysglycaemia

Poverty is a barrier to healthcare, and conversely, poor health can lead to loss of wages and result in a negative feedback loop called the health-poverty trap (Dhullar 2018; Bank 2014). Efficient allocation of resources is key in managing the dual disease entity of dysglycaemia and TB. To the best of our knowledge, there is no body of work that has jointly quantified the cost of care for the two disease entities in Kenya. Separately, TB cost survey in Kenya estimated the costs from a patient perspective and found the cumulative costs of drug-sensitive TB and drug-resistant TB to be USD 261 versus USD 1452, respectively (Kirubi et al. 2021).

The WHO has designed a STEPwise Approach to NCD Risk Factor Surveillance abbreviated as STEPS. The non-communicable disease (NCD) STEP-survey done in Kenya in 2015 estimated the incidence of DM to be 2.4% and on the other hand 3.1% of Kenya's population having impaired fasting glycaemia (Wamai et al. 2018).

The magnitude of DM and prediabetes in the TB population in Kenya is, however, higher than the national average given the association between the two disease entities (Owiti et al. 2017; Amayo and Mutai 2018).

Though the TB care is free in Kenya, the costs during treatment impose a significant financial burden on patients and their households (Kirubi et al. 2021).

Kairu et al. also did work from the health system perspective and found that the average cost for passive case-finding (PCF) for TB is US$38 and US$60, using the bottom up (BU) and top-down (TD) economic approaches, respectively. Using both approaches, they also generated the average cost of a first-line treatment—6-month (FLT) course. They factored in both diagnostics and follow-up and costed it at US$135 and US$160, respectively. In the case of drug-resistant TB (DR-TB), treatment was approximately KES 323,000 (US$3,230.28) and KES 392,652 (US$3,926.52) for the 9-month short regimen (Kairu et al. 2021).

On the other hand, the patient-based costs (for diabetes only) have also been estimated. The mean annual direct patient cost was KES 53,907 or US$ 528.5 (Oyando et al. 2020). This amount did not factor in the indirect costs, meaning the actual total cost is higher. Overall, the high incidence of catastrophic costs (63.1%) was found to be higher in the DM patients who presented with hypertension as a comorbidity which increased even further to 75.4% when transport costs were added (Oyando et al. 2020).

### Funding situation of LICs and LMICs

Globally, funding for treatment, prevention and diagnosis of TB has not reached target but healthcare expenditure for TB rose by 2020. The unprecedented COVID-19 pandemic did not help matters that final monies allocated were lower due to reallocation of funding for the COVID-19 response. In terms of research and development (R&D), funding for TB research has fallen below less than half of target with only USD 906million allocated to TB research by 2020 in lieu of the projected USD 2 billion. With the exception of the BRICS country block, i.e. Brazil, Russian Federation, India, China and South Africa), most low to low/middle income (LMCs and LMICs) are still heavily reliant on international donor funding (MacNeil et al. 2020). Kenya being an LMIC is this category (World Bank GDP 2022). Vision 2030 aims to transform Kenya into a ‘newly industrializing, middle-income country providing high quality of life to all its citizens by 2030’. Given this ambitious idea, we need to efficiently tackle resource allocation and consider strategies to reduce financial burden, provide social protection and enhance testing for and treatment for the dysglycaemia spectrum.

### Ultimate progress on END TB 2035

Substantial gaps remain from a research and policy perspective that need to be addressed if we are to achieve the END TB 2035 goal. The authors of the Global TB reports in the past 3 years themselves write that the strategy and goals attached to it are ambitious. Catastrophic costs as at 2020 still affect almost half of the population afflicted by TB while the goal was to be at zero (MacNeil et al. 2020). Closer to home in Kenya, based on the TB patient-costs survey, TB-related payments led to an increase in the proportion of patients living below the poverty line from 13.9% to 31.1%. This brings us back to the negative feedback loop of the poverty-health care trap. To expound more on this socio-economic hardship, 62.5% lost jobs due to TB and some coping mechanisms included sale of assets, clearing savings and taking credit (). These costs do not include the costs of any associated dysglycaemia which costs a pretty penny to treat. With social protection, can these figures be reduced? Nutrition is one of the five important determinants of TB according to the Global TB Report of 2020, and body mass index (BMI) is an important aspect of this and a strong correlate of diabetes in TB (Alkabab et al. 2021; MacNeil et al. 2020; Owiti et al. 2017; Wachinou et al. 2021). Given that only 39% of the TB case notifications recorded in 2021 within Kenya had a normal BMI, we need to urgently address screening for dysglycaemia alongside markers that are easy to measure and record like BMI (Program-NTLLD-P NTLaLD 2021). Effective TB vaccines are an urgent need and several candidates are in the pipeline (MacNeil et al. 2020), a promising one being the M72 /ASO1e which provided 3 year- protection against progression to PTB for at least 3 years (Tait et al. 2019; Meeren et al. 2018).

### 'Authors' critical views'/recommendations

As a country, we can consider going upstream to dig root cause by investing in dysglycaemia testing in lieu of waiting downstream for the trickle-down effect of the undiagnosed and unmanaged dysglycaemia.

Mapping of the dysglycaemia belt in Kenya may be a critical activity to be undertaken in our country to ensure targeted resource allocation in the counties.

Increase funding allocated for both TB and dysglycaemia in the counties.

Strengthening capacity of research centres, primary care hospitals included, should also be a priority in terms of training of health care workers in research, equipping with basic machines for testing for blood sugar and monitoring. Implementing targeted screening strategies for PWDM since there is a one-and-a-half-fold increase in risk of acquiring latent tuberculosis infection (LTBI) in this population.

Social protection to limit or prevent the catastrophic costs associated with both TB, TB/HIV co-infection coupled with additional cost of catering to dysglycaemia should be a consideration. The national payor should consider subsidizing the additional costs of dysglycaemia care like oral and injectable glucose lowering agents. Improving access to insulin and insulin syringes as well as access to testing at non-tertiary facilities in a consistent fashion.

Strengthening patient advocacy organizations which do pooling of resources to purchase insulin syringes in a cooperative society model can subsidize care for patients.

Nutritional counselling for TB patients with dysglycaemia should also have more emphasis and not be reserved for those with malnutrition only.

Practical, abbreviated cardiometabolic disease guidelines that can be understood by primary care health workers in TB and HIV care facilities are needed to help with choice of medication if a patient presents with TB, TB/HIV co-infection and dysglycaemia.

## Conclusions

Multisectoral collaboration is key to achieving END TB 2035; the Kenya Diabetes Study Group (KDSG), Kenya Association of Physicians (KAP), the Non-Communicable Disease Alliance of Kenya (NCDAK) and the National TB Leprosy and Lung Treatment Program (NTLLDP) as well as the National Hospital Insurance Fund (NHIF) need to forge stronger alliances to tackle the issue of bi-directional testing, research and subsidized care for TB and the dysglycaemia syndemic. Given the high TB and HIV co-infection rate in Kenya, more home-grown research to establish the effect of HIV on this syndemic is needed since current literature is inconclusive.

## Data Availability

Not applicable.

## Abbreviations

BRICS: Brazil, Russian Federation, India, China, and South Africa
BMI: Body mass index
BU: Bottom-up costing approach
COVID-19: Coronavirus Disease- 2019
CVA: Cerebrovascular Accident
CXR: Chest Radiograph
DALYs: Disability Adjusted Life Years
DM: Diabetes Mellitus
DR-TB: Drug-resistant tuberculosis
FLT: First-line treatment
FPG: Fasting plasma glucose
HbA1C: Glycated haemoglobin
IHD: Ischaemic heart disease
KAP: Kenya Association of Physicians
KDSG: Kenya Diabetes Study Group
LICs: Low-income countries
LMICs: Low/middle-income Countries
NTLLDP: National Tuberculosis Leprosy and Lung Treatment Program
NTP: National Tuberculosis Program
NCD: Non-communicable diseases
NCDAK: Non-Communicable Disease Alliance of Kenya
PCF: Passive case finding
PTLD: Post-tuberculosis lung disease
RBS: Random blood sugar
STEPS: STEPwise Approach to NCD Risk Factor Surveillance
TD: Top- down costing approach
T1D: Type 1 Diabetes
T2D: Type 2 Diabetes
UHC: Universal health coverage
WHO: World Health Organization
